# One-Step Synthesis of Silver Nanoparticles on Polydopamine-Coated Sericin/Polyvinyl Alcohol Composite Films for Potential Antimicrobial Applications

**DOI:** 10.3390/molecules22050721

**Published:** 2017-04-30

**Authors:** Rui Cai, Gang Tao, Huawei He, Kai Song, Hua Zuo, Wenchao Jiang, Yejing Wang

**Affiliations:** 1 College of Biotechnology, Southwest University, Beibei, Chongqing 400715, China; cairui0330@email.swu.edu.cn; 2 State Key Laboratory of Silkworm Genome Biology, Southwest University, Beibei, Chongqing 400715, China; taogang@email.swu.edu.cn (G.T.); hehuawei@swu.edu.cn (H.H.); 18728301293@163.com (K.S.); jwc1245@163.com (W.J.); 3 College of Pharmaceutical Sciences, Southwest University, Beibei, Chongqing 400715, China; zuohua@swu.edu.cn

**Keywords:** sericin, polyvinyl alcohol, polydopamine, silver nanoparticles, one-step synthesis, antibacterial activity

## Abstract

Silk sericin has great potential as a biomaterial for biomedical applications due to its good hydrophilicity, reactivity, and biodegradability. To develop multifunctional sericin materials for potential antibacterial application, a one-step synthesis method for preparing silver nanoparticles (AgNPs) modified on polydopamine-coated sericin/polyvinyl alcohol (PVA) composite films was developed. Polydopamine (PDA) acted as both metal ion chelating and reducing agent to synthesize AgNPs in situ on the sericin/PVA composite film. Scanning electron microscopy and energy dispersive spectroscopy analysis revealed that polydopamine could effectively facilitate the high-density growth of AgNPs as a 3-D matrix. X-ray diffractometry studies suggested the synthesized AgNPs formed good face-centered cubic crystalline structures. Contact angle measurement and mechanical test indicated AgNPs modified PDA-sericin/PVA composite film had good hydrophilicity and mechanical property. The bacterial growth curve and inhibition zone assays showed the AgNPs modified PDA-sericin/PVA composite film had long-term antibacterial activities. This work develops a new method for the preparation of AgNPs modified PDA-sericin/PVA film with good hydrophilicity, mechanical performance and antibacterial activities for the potential antimicrobial application in biomedicine.

## 1. Introduction

Silk sericin (SS) is a natural macromolecular protein from silkworm cocoon, which makes up 25% of the total silk protein [1]. It is produced in the middle silk gland [2]. Sericin protein consists of 18 amino acids, including some essential amino acids [3]. Serine is the main part of human skin natural moisture factor (NMF) [4]. The content of serine in sericin is approximately 33.43% [5]. Thus, sericin is an excellent moisturizing agent [6]. As an inexpensive, readily available, biodegradable, and biocompatible substance [7,8,9], sericin has attracted increasing interest in the field of biomaterials. Furthermore, sericin has the mitogenic effect on mammalian cells, which places it in a favorable position for tissue engineering [10,11], especially for the growth and migration of keratinocytes and fibroblasts [12,13]. As sericin can promote collagen formation in wounds and thereby accelerate the re-epithelialization of open wounds, sericin has great potential as a wound healing agent [14]. However, sericin has high levels of random coil structures [15,16], which makes it behave like an amorphous material [17] and be fragile in a dry state. Cross-linking, blending, or copolymerization with other substances could overcome the brittleness of sericin [18]. Previously, we blended sericin with polyvinyl alcohol (PVA) to prepare a SS/PVA composite film, which had good hydrophilicity and mechanical properties with potential in wound healing applications. However, silk sericin/polyvinyl alcohol film (SS/PVA) itself lacks antibacterial ability which is not favorable for wound healing because wound infection is an important factor affecting wound healing. There is increasing interest in the development of antifouling or antimicrobial films, which will enhance the application of biological materials [19]. Thus, the development of SS/PVA film with antibacterial ability is required for the application of sericin in the field of wound healing.

Surface immobilization of silver nanoparticles (AgNPs) is a popular method to endow materials with antimicrobial ability because AgNPs have broad-spectrum antimicrobial activities [20,21,22,23,24,25,26], low cytotoxicity [27,28] and anti-inflammatory characteristics [29]. AgNPs has a large specific surface area, thereby greatly increasing the probability of AgNPs contact with microbial surfaces. In addition, this rarely leads to the development of resistant microbes. Therefore, AgNP is a very important antibacterial agent [30,31,32]. In the early stages, AgNPs are pre-synthesized first by some physical or chemical method and then immobilized on the surface of a material via physical or chemical adsorption. Since these procedures are quite complicated and tedious, this strategy has been gradually abandoned [33,34,35]. As a safe and green method, the use of biomolecules to prepare AgNPs has received more and more attention as it may have a better biocompatibility [36]. However, the biosynthesized AgNPs cannot be directly modified on the surface of the material. Therefore, as an effective alternative, the synthesis of AgNPs in situ on the material surface has been developed. UV irradiation reduction is one of the most convenient methods for the synthesis of AgNPs in situ [37], however, due to the small number of active sites on the SS/PVA film surface, the amounts of AgNPs that can be synthesized on the surface are limited. Various polymers such as polyamide network polymer, poly(vinyl pyrrolidone) and polyacrylic acid have been developed as a 3-dimensional (3D) matrix with a layer-by-layer assembly technique to provide more Ag^+^ binding sites for the high-density growth of AgNPs [38], but the multi-step procedures of this technique are too complicated. Hence, developing a practical one-step synthesis of AgNPs is necessary for antimicrobial surface modification.

Polydopamine (PDA) is a hormone and neurotransmitter in the body [39]. It has strong adsorbability and is environment-friendly, thus it is often used as a versatile platform for secondary reactions after coating on a matrix, which could serve as a carrier to absorb other substances [40]. Based on the self-polymerization of dopamine under weak alkaline conditions, PDA can adhere to almost all material surfaces [41,42]. Since dopamine has metal ion chelating ability and redox activity [43,44], it could directly reduce the adsorbed Ag^+^ on the dopamine-coated material surface to form AgNPs via a one-step reaction [45,46]. These properties may facilitate the use of PDA as a 3D matrix to enhance the adsorption of Ag^+^ and promote the high-density growth of AgNPs. In addition, the presence of PDA could avoid direct exposure of AgNPs to oxygen and slow down the release of silver ions. Therefore, a PDA coating on sericin composite films surface might enhance the capacity of AgNPs and produce better antibacterial activity with sericin films.

**Figure 1:**
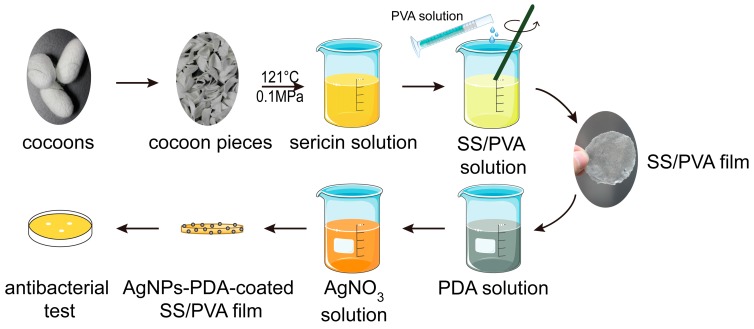
The flow diagram illustrates the preparation and antimicrobial analysis of AgNPs-PDA-SS/PVA composite film.

In this work, we have established a one-step reduction method to prepare AgNPs-modified PDA-SS/PVA films (Figure 1). PDA was coated on the SS/PVA composite film and then used to adsorb and reduce Ag^+^ to form AgNPs. The prepared composite films were characterized by scanning electron microscopy (SEM), energy dispersive spectroscopy (EDS), X-ray diffractometry (XRD) and fourier transfer infrared spectroscopy (FT-IR) to analyze the PDA coating and the synthesis of AgNPs. Antimicrobial assays were conducted to investigate the antimicrobial activity of AgNPs-modified PDA-SS/PVA film against *Escherichia coli* (*E. coli*) and *Staphylococcus aureus* (*S. aureus*). The AgNPs-modified sericin/PVA composite films exhibited good bactericidal activity and long-lasting antimicrobial performance. This prepared AgNPs-PDA-SS/PVA composite film may have potential applications in biomedical materials such as wound dressings.

## 2. Results and Discussion

### 2.1. Preparation of PDA Coated SS/PVA Film

**Figure 2:**
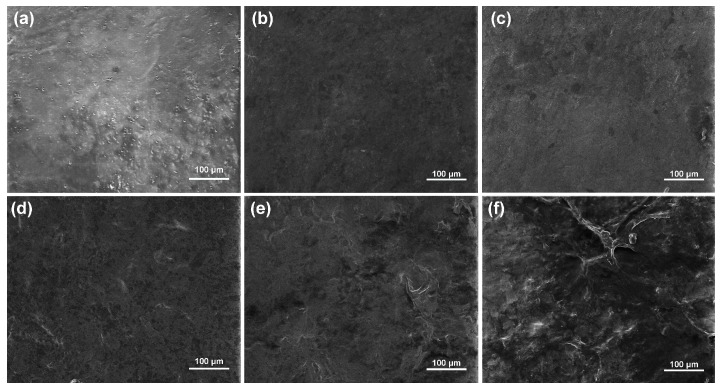
SEM images of SS/PVA film (**a**) and PDA-coated SS/PVA films prepared with dopamine concentration of 0.5 (**b**), 1.0 (**c**), 2.0 (**d**), 3.0 (**e**) and 5.0 mg/mL (**f**), respectively.

The adsorption of PDA on the film surface is affected by monomer concentration, reaction temperature and time. To determine the best condition for forming a uniform PDA film, SEM experiments were carried out to observe the surface morphologies of SS/PVA film and PDA-coated SS/PVA film prepared with different dopamine concentrations (Figure 2).

SS/PVA film had a smooth surface, suggesting the cross-linking of sericin and PVA was successful (Figure 2a). Smooth surfaces with some insignificant tiny dots and pores could be observed on SS/PVA films treated with 0.5–2.0 mg/mL dopamine solutions (Figure 2b–d)). Rough surfaces with obvious protuberances could be observed on SS/PVA films treated with 3.0 and 5.0 mg/mL dopamine solution (Figure 2e–f). The results showed that PDA could coat on the composite films with dopamine concentrations as low as 0.5 mg/mL. The degree of PDA adsorption is key for the synthesis and growth of AgNPs. A low concentration of dopamine will form a thin PDA film, which may be not favorable for the high-density growth of AgNPs. However, high concentrations of dopamine will form a thick PDA film, which may affect the flexibility of the material. Hence, the dopamine concentration was selected as 2.0 mg/mL, and the surface of PDA-coated SS/PVA film was uniform without obvious protuberances. Besides the concentration of dopamine, reaction temperature and time also affected the adsorption of PDA on the film surface. A previous study shows that high temperature and agitation could speed up the formation of PDA films [47]. Long deposition times could also lead to the formation of a thick PDA film [48]. Thus, in this study, the modification of PDA on the SS/PVA film surface was carried out under constant stirring for 12 h at room temperature in 2.0 mg/mL dopamine.

### 2.2. FESEM, EDS and XRD Analysis

**Figure 3:**
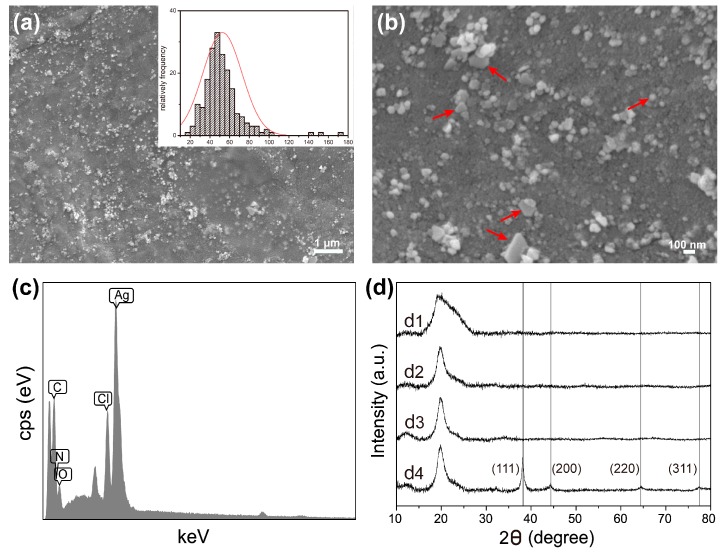
Field emission scanning electron microscope images of AgNPs-modified PDA-SS/PVA film (**a**) and (**b**). Inset in (a), particle size distribution of AgNPs. The red arrows in (b) indicated the different morphologies of the synthesized AgNPs. (**c**) EDS spectrum of a selected area of AgNPs-PDA-SS/PVA film. (**d**) XRD patterns of pure sericin (d1), SS/PVA film (d2), PDA-SS/PVA film (d3) and AgNPs modified PDA-SS/PVA film (d4).

Since PDA could adsorb Ag^+^ and catechol moiety of PDA could efficiently reduce Ag^+^ to AgNPs by itself, no additional reagents or treatments are involved in the synthesis of AgNPs. As shown in Figure 3a,b, the surface of the SS/PVA composite films were covered by high-density and uniformly distributed nanoparticles. The size of nanoparticles was mainly in the range of 35–65 nm. The synthesized nanoparticles showed different morphologies, such as spheres, triangles and hexagons (Figure 3b). The EDS spectrum of the modified composite film showed a well-defined peak corresponding to silver, indicating the existence of this element on the modified SS/PVA film (Figure 3c). The carbon, nitrogen, oxygen and chlorine peaks were attributed to sericin, PVA and PDA. The XRD patterns of sericin, SS/PVA film, PDA-SS/PVA film and AgNPs-modified PDA-SS/PVA film were shown in Figure 3d. A broad peak located at 2θ = 19.2° was observed in the sericin pattern, which was consistent with a previous report [49]. A characteristic broad peak located at 2θ = 19.8° was observed in the other XRD patterns, which may be attributed to the crystalline diffraction of PVA. No significant change was observed on the peaks after PDA coating, suggesting that PDA coating did not affect the crystal structure of SS/PVA composite film. For the AgNPs-modified PDA-SS/PVA film, four more peaks were observed at 2θ = 38.12°, 44.33°, 64.46° and 77.41°, which could be assigned to the crystal planes (111), (200), (220) and (311) of the face-centered cubic structure of the synthesized AgNPs, respectively [50,51].

The results suggested that the synthesized AgNPs formed good crystalline structures. The high-density, good crystallinity and uniform distribution of AgNPs should greatly enhance the antibacterial activity of SS/PVA film.

### 2.3. FT-IR Analysis

**Figure 4:**
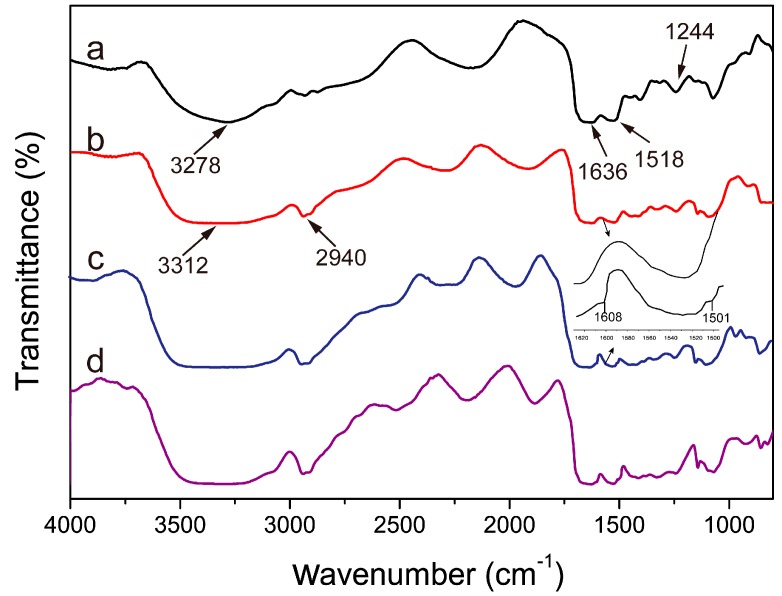
FT-IR spectra of pure sericin (**a**), SS/PVA composite film (**b**), PDA-SS/PVA composite film (**c**) and AgNPs-modified PDA-SS/PVA composite film (**d**).

The structures of sericin, SS/PVA composite film, PDA-SS/PVA composite film and AgNPs-modified PDA-SS/PVA composite film were characterized by fourier transform infrared spectroscopy. As shown in Figure 4, three characteristic peaks located at 1636, 1518 and 1244 cm^−1^ were observed in all the FT-IR spectra, corresponding to the amide I, II and III bands of sericin, respectively [52]. After PDA coating, two more weak peaks were observed at 1608 and 1503 cm^−1^. The former could be attributed to the C=C stretching and N-H deformation vibration of the indole or indoline structures in PDA [53]. The latter may be due to the skeletal vibrations of the PDA benzene ring [54]. The result indicated that PDA was successfully coated on the surface of SS/PVA film, which was in line with the SEM test results. PDA coating and AgNPs modification had no effect on the amide peaks of sericin, indicating these processes did not affect the structure of the SS/PVA film.

### 2.4. Wettability and Water Uptake Ability Measurements

**Figure 5:**
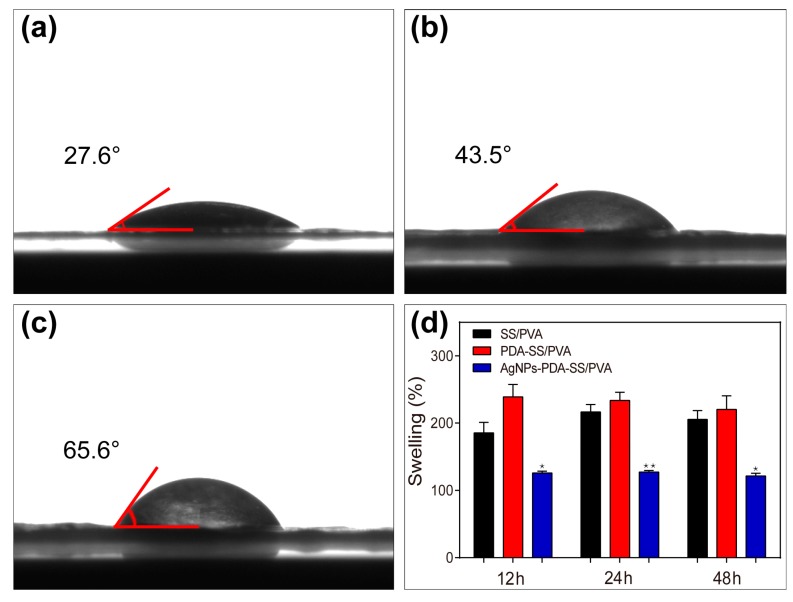
Water contact angles of SS/PVA (**a**), PDA-SS/PVA (**b**), and AgNPs-PDA-SS/PVA (**c**). Swelling ratio of these films (**d**) (n = 3 per group; * indicates significant differences compared with SS/PVA film at *p* < 0.05, ** indicates significant differences compared with SS/PVA film at *p* < 0.01).

The water contact angles of SS/PVA, PDA-SS/PVA and AgNPs-PDA-SS/PVA films were shown in Figure 5a–c, respectively. The results showed the water contact angle of SS/PVA film was 27.6°, suggesting it was highly hydrophilic. The water contact angle of PDA-SS/PVA film was 43.5°, indicating the surface of PDA-SS/PVA film was hydrophilic. After AgNP modification, the water contact angle increased to 65.6°, meaning the surface still kept a certain degree of hydrophilicity. This may be due to the fact most of the surface hydrophilic groups were covered by the evenly distributed AgNPs.

To determine the water absorption of the prepared films, the swelling property of these films were analyzed, as shown in Figure 5d. The swelling ratios of SS/PVA and PDA-SS/PVA films were about twice as large as the control after the samples were immersed in PBS buffer (pH 7.4) for 12 h, 24 h and 48 h, indicating these films had a better hygroscopicity. The swelling ratio of AgNPs-PDA-SS/PVA film was about 1.2 times of the control, suggesting AgNPs-PDA-SS/PVA film had good hygroscopicity. The effect of AgNPs modification on the SS/PVA film may be attributed to the hydrophilic sites being massively occupied by AgNPs. The swelling ratios did not change significantly with increased time. The reason may be the total surface area of the two-dimensional film interacted with water molecules during the initial swelling stage. The results suggested that AgNPs-modified PDA-SS/PVA film had good hydrophilicity and hygroscopicity, which may be useful in the field of wound dressings to maintain the wetness of the wound and reduce secondary damage.

### 2.5. Mechanical Properties

**Figure 6:**
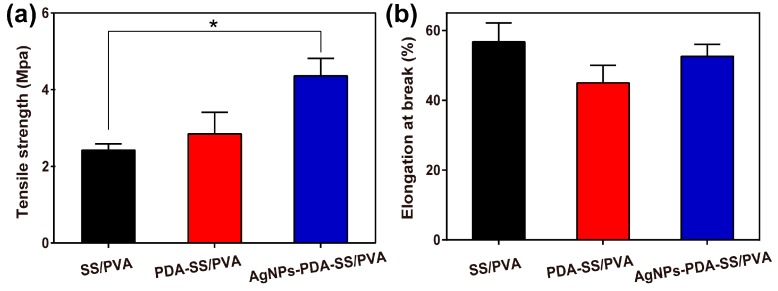
Mechanical properties of the films: (**a**) tensile strength and (**b**) elongation at break (n = 3 per group; * indicates *p* < 0.05).

The tensile strength and elongation at break of SS/PVA, PDA-SS/PVA and AgNPs-PDA-SS/PVA films were shown in Figure 6. Tensile testing showed that PDA-SS/PVA and AgNPs-PDA-SS/PVA films exhibited a higher tensile strength compared to SS/PVA film. The enhancement of tensile strength may be attributed to the increase of film thickness after surface modification with AgNPs. The elongation value at break represents the flexibility of the film [55]. After PDA coating and AgNPs modification, no significant changes were observed in the elongation at break. These results indicated that AgNPs-PDA-SS/PVA film was stronger and tougher than SS/PVA film, which may have a potential application in biomedical materials such as wound dressing and skin replacement, since it had a typical tensile strength in the range of 2.5–16 Mpa [56,57,58].

### 2.6. Inhibition Zone Assays

**Figure 7:**
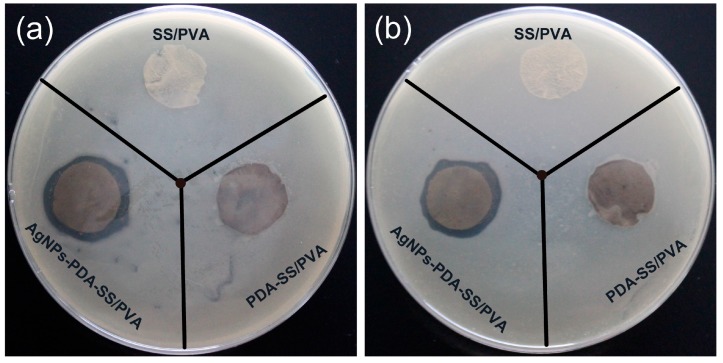
Inhibition zone assays of SS/PVA, PDA-SS/PVA, AgNPs-PDA-SS/PVA against *E. coli* (**a**) and *S. aureus* (**b**).

The anti-bactericidal activities of SS/PVA, PDA-SS/PVA and AgNPs-PDA-SS/PVA films were evaluated against Gram-negative bacteria (*E. coli*) and Gram-positive bacteria (*S. aureus*), respectively. As shown in Figure 7, no significant inhibition zones were observed for SS/PVA and PDA-SS/PVA films. However, after AgNP modification, the film could efficiently kill the surrounding microbes, and form evident inhibition zones, indicating the antibacterial activity of the film depended on the presence of AgNPs. The diameters of the inhibition zones were summarized in Table 1. It was noted that the inhibition effect of AgNPs-PDA-SS/PVA film against Gram-positive bacteria was not as significant as that against Gram-negative bacteria, which may be due to the fact that Gram-positive bacteria is encased in a plasma membrane covered with a thick layer of peptidoglycan (about 20–80 nm) that limits the penetration of silver nanoparticles [59]. The result suggested the prepared AgNPs-PDA-SS/PVA film significantly inhibited the growth of *E. coli* (a) or *S. aureus* (b).

### 2.7. Bacterial Growth Curve

**Figure 8:**
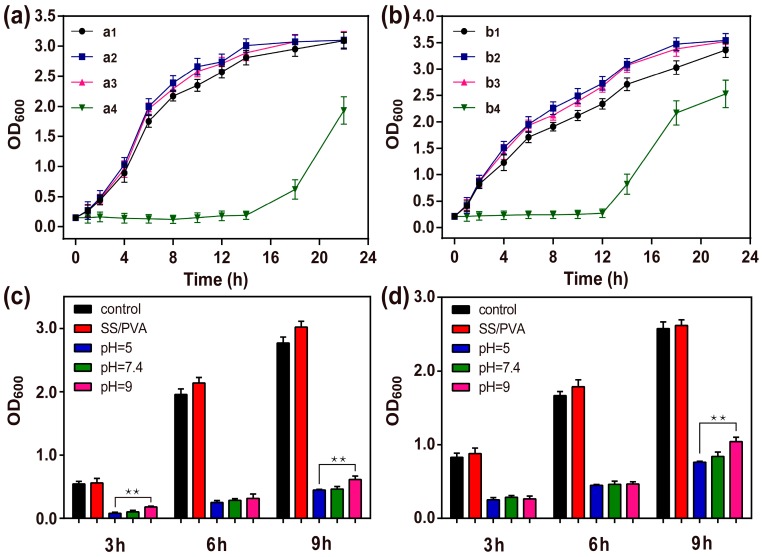
Growth curves of *E. coli* (**a**) and *S. aureus* (**b**). Bacteria without treatment (a1, b1); Bacteria treated with SS/PVA films (a2, b2), PDA-SS/PVA films (a3, b3) and AgNPs-PDA-SS/PVA films (a4, b4). Long-term bactericidal activity against *E. coli* (**c**) and *S. aureus* (**d**) (n = 3 per group; ** indicates *p* < 0.01).

To further evaluate the antimicrobial ability of AgNPs-PDA-SS/PVA film, the bacterial growth curves were analyzed by measuring the optical density (OD) of bacteria at 600 nm, as shown in Figure 8a, b. SS/PVA and PDA-SS/PVA films showed negligible antibacterial activity against *E. coli* and *S. aureus* compared with the control. However, after AgNP modification on the surface of SS/PVA film, the lag phase of *E. coli* and *S. aureus* was significantly prolonged to 14 h and 12 h, respectively. The result indicated AgNPs-PDA-SS/PVA film had good bacteria-killing and bacterial infection-inhibiting ability. The inhibitory effect of AgNPs-PDA-SS/PVA film against Gram-positive bacteria was less than that against Gram-negative bacteria, which was in good agreement with the inhibition zone assay results. It was noted that the bacterial growth could recover after 14 h or 12 h, which may be attributed to the adaptability of bacteria to Ag^+^. In the real world, the bacteria growth conditions are relatively poor, hence, the prepared AgNPs-PDA-SS/PVA film may show better performance in antibacterial biomedicine applications.

### 2.8. Long-term Antimicrobial Stability Analyze

To valuate the long-term antimicrobial stability, the antimicrobial activities of AgNPs-PDA-SS/PVA film against *E. coli* and *S. aureus* after treatment with different pH solutions for 24 h were measured. As shown in Figure 8c, d, even after 9 h had passed, AgNPs-PDA-SS/PVA film still showed a strong antibacterial activity compared to the control, especially under acidic conditions. The reason may be attributed rather to the passivation of AgNPs. The polymer film is more stable under acidic conditions than that under other conditions, which reduces the loss of AgNPs. The result indicated that AgNPs were stable and AgNPs-PDA-SS/PVA film had a long-term and stable antibacterial activity.

### 2.9. Mass Loss Studies

**Figure 9:**
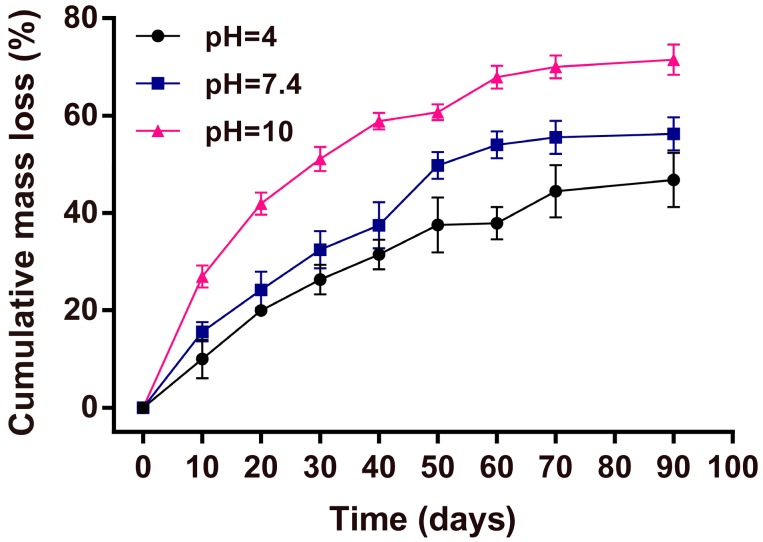
Mass loss kinetics of AgNPs-PDA-SS/PVA films at different pH values.

The stability of wound dressing is directly related to its performance. To assess the potential of AgNPs-PDA-SS/PVA film as a wound dressing, we investigated the stability of AgNPs-PDA-SS/ PVA films at different pH values. The mass losses of the films at pH 4, 7.4 and 10 were shown in Figure 9. The results showed that the mass loss increased with time. Under alkaline conditions (pH 10), the mass loss of AgNPs-PDA-SS/PVA film was about 71% after 90 days of treatment. Under neutral conditions (pH 7.4), the mass loss was about 50%. Under acidic conditions (pH 4), approximately 46% of the weight was lost. It was noted the mass loss of AgNPs-PDA-SS/PVA film under alkaline conditions was faster than that under an acidic or a neutral condition. This may be because in sericin the content of acidic amino acids (Glu and Asp, 24%) was higher than that of alkaline amino acids (Lys, His and Arg, 8%) [60], and the isoelectric point of sericin is approximately 3.8 [61]. In addition, PVA shows weak acidity in water. The result suggested that AgNPs-PDA-SS/PVA films had good stability and were environmentally friendly, because the film could be slowly degraded in a wet environment.

## 3. Materials and Methods

### 3.1. Materials

Silkworm cocoons were supplied by the State Key Laboratory of Silkworm Genome Biology, Southwest University (Beibei, Chongqing, China). Dopamine hydrochloride and silver nitrate (AgNO_3_, AR, 99.99%) were purchased from Aladdin Corp. (Shanghai, China). Tris(hydroxymethyl)aminomethane (Tris) and hydrochloric acid (HCl) were purchased from Sangon Biotech (Shanghai, China). MiliQ water made by a MilliQ water purification system (Millipore, Billerica, MA, USA) was used in the experiments.

### 3.2. Preparation of AgNPs Modified PDA-SS/PVA Composite Film

Sericin was extracted from silk cocoons according to a previously reported procedure with minor modifications [62]. PVA was dissolved under a constant stirring speed at 80°C until it was fully dissolved to a final concentration of 5% (*w*/*t*). Then 4% (*w*/*t*) sericin solution and 5% (*w*/*t*) PVA solution were mixed together with a 1:1 ratio at 60°C for at least 30 min, and then frozen under −20 °C and thawed at room temperature for four cycles to form a SS/PVA hydrogel. The hydrogel became SS/PVA blend film upon drying. Dopamine powder was dissolved in Tris-HCl buffer to formulate different concentrations (0.5, 1.0, 2.0, 3.0 and 5.0 mg/mL), and then adjusted to pH 8.5. SS/PVA films were immersed directly into the freshly prepared dopamine solutions for 12 h with continuously magnetic stirring at 37 °C. Then the PDA coated SS/PVA films were taken out and rinsed with MiliQ water until the washing water became clear. Furthermore, the prepared films were dried at room temperature and then soaked in 30 mM AgNO_3_ at room temperature for 12 h. Finally, the AgNPs modified PDA-SS/PVA composite films were produced after repeated washing and drying at room temperature.

### 3.3. Materials Characterization

The morphologies of PDA-SS/PVA film and AgNPs modified PDA-SS/PVA film were imaged on a JCM-5000 SEM instrument (JEOL, Tokyo, Japan). At the same time, energy-dispersive X-ray spectroscopy (EDS) (INCA X-Max 250, Oxford, England) were collected to analyze the chemical elements in these samples. The modified and unmodified AgNPs SS/PVA films were measured on an X’Pert Powder XRD with a 2θ range of 10° to 80° (PANalytical, Almelo, The Netherlands). FT-IR measurements were performed in the wavenumber of 4000–800 cm^−1^ with 2 cm^−1^ resolution on a Nicolet iz10 FT-IR spectrometer (ThermoFisher Scientific Corp, Waltham, MA, USA).

### 3.4. Wettability Measurement

The wettabilities of the samples were measured via sessile drop contact angle measurements on a KRÜSSDSA100 contact angle system (Krüss, Hamburg, Germany) at room temperature. A sample was examined in five different positions. A water droplet of 4 μL was dropped on the surface of the sample, and then the contact angle was measured.

### 3.5. Water Uptake Ability

The swelling property of the composite film in water was investigated by the gravimetric method developed by Mandal et al. [63] with a minor modification. The dried composite films were weighed as *W_1_* and immersed into 10 mL water at 37 °C. At various time intervals, the films were taken out, and extra water on the surface of the films was carefully removed with filter paper, then weighed as *W_2_* immediately. The swelling ratio (*S*) was calculated according to the following equation:

*S* (%) = (*W*_2_ − *W*_1_) × 100%/*W_1_* (1)

At least three repeats were performed for each film under the same condition and the average value was used to evaluate the swellability of the prepared films.

### 3.6. Mechanical Analysis

The mechanical property of composite film was measured on an AG-Xplus universal testing machine (Shimadzu, Kyoto, Japan) equipped with a 1000-N load cell at a crosshead speed of 3 mm/min. Each composite film was tested with a dimension of 4 cm × 1 cm (length × width). The thickness of the composite film was determined by SEM. The raw data were transformed into true stress (σ) and strain (ε) to plot the stress–strain curves [64].

### 3.7. Inhibition Zone Assay

The inhibition zone assay was performed according to the method described by Schillinger and Lucke [65]. *E. coli* and *S. aureus* were inoculated into 100 mL LB medium (pH 7.4) at 37 °C under a constant shaking speed of 220 rpm. Bacteria at lag phase were applied to agar medium plates in the presence of circular AgNPs modified or unmodified sericin composite films (d = 1.50 cm). After incubation at 37 °C overnight, the antibacterial activities of the samples were evaluated according to the diameters of bacterial inhibition zones.

### 3.8. Growth Curve Assay

The bacterial growth curve assay was carried out according to Pal’s protocol [66]. Bacteria at lag phase were inoculated into 10 mL LB medium (pH 7.4) and cultured under a constant shaking speed of 220 rpm at 37 °C in the presence of AgNPs modified or unmodified SS/PVA blend films (1 cm × 1 cm). About 0.5 mL bacterial suspensions were collected at different time intervals to measure the optical density of bacteria at 600 nm (OD_600_). All growth curve tests were made in triplicate to ensure the reproducibility of the tests.

### 3.9. Long-Term Antimicrobial Stability Test

For long-term antimicrobial stability test, AgNPs-modified PDA-SS/PVA films (1 cm × 1 cm) were immersed in PBS buffer with different pH (5, 7.4 or 9) for 24 h, respectively. SS/PVA blend film treated with bacterial was set as a positive control. At various time intervals, the antimicrobial activity test was performed against *S. aureus* and *E. coli* using the previously described method.

### 3.10. Mass Loss Test

The AgNPs-modified sericin composite films (3 cm × 3 cm) were soaked into PBS with different pH values (4, 7.4, 10) at 37 °C. PBS was replaced daily. At given time points, the films were taken out, washed, lyophilized, and weighed. The mass loss ratio was calculated as the difference between the initial and following dry masses at indicated times divided by the initial dry mass.

### 3.11. Statistics

All experiments were performed in triplicate, and data were presented as the means ± SD. The statistical significance was determined by paired and unpaired Student’s *t*-tests together with ANOVA (* *p* < 0.05, ** *p* < 0.01).

## 4. Conclusions

In this study, we have developed a one-step method to prepare AgNPs-PDA-SS/PVA film without the introduction of additional reduction reagents. High-density AgNPs are synthesized on the surface of SS/PVA film via the adsorption and direct reduction of PDA. The synthesized AgNPs are evenly distributed and have good crystallinity. The PDA coating and AgNPs modification do not alter the structure of the SS/PVA film. The prepared AgNPs-PDA-SS/PVA film has good hydrophilicity, hygroscopicity, and mechanical performance. More importantly, it has a long-term stable antibacterial activity against both Gram-negative and Gram-positive bacteria. In addition, the film has good stability and is environmentally friendly. This film with various excellent performances is expected to be useful in biomaterial applications such as wound dressings. Furthermore, a simple procedure is proposed to introduce dopamine coating on film preparations, which will facilitate the fabrication of more dopamine-containing compound films or nanofilms, which may be a productive research area in the future.

## Figures and Tables

**Table 1:** Diameters of inhibition zones of PDA-SS/PVA film and AgNPs-PDA-SS/PVA film against *E. coli* and *S. aureus*.

Bacteria	Control (cm)	PDA-SS/PVA (cm)	AgNPs-PDA-SS/PVA (cm)
*E. coil*	1.55 ± 0.10	1.65 ± 0.11	2.03 ± 0.08
*S. aureus*	1.55 ± 0.15	1.62 ± 0.06	1.86 ± 0.06
